# Laboratory Genetic Testing in Clinical Practice 2014 

**DOI:** 10.1155/2015/574798

**Published:** 2015-03-24

**Authors:** Ozgur Cogulu, Jacqueline Schoumans, Gokce Toruner, Urszula Demkow, Emin Karaca, Asude Alpman Durmaz

**Affiliations:** ^1^Department of Medical Genetics, Faculty of Medicine, Ege University, 35100 Izmir, Turkey; ^2^Cancer Cytogenetic Unit, Department of Medical Genetics, Lausanne University Hospital, 1011 Lausanne, Switzerland; ^3^Institute of Genomic Medicine, UMDNJ-NJ Medical School, Newark, NJ 07103, USA; ^4^Department of Laboratory Diagnostics and Clinical Immunology, Medical University of Warsaw, 00-576 Warsaw, Poland

The laboratory genetic testing is rapidly emerging in clinical practice. Recently, in parallel with the report of human genome project, next generation sequencing techniques are widely applied to study human genome providing a basis for integrating information about genome, exome, and transcriptome of a living organism. The future applications of genetic testing are discussed in popular media everyday. Accordingly, this special issue was prepared to provide a basis for integrating information from this emerging field. Among them, we have given place to research articles as well as clinical studies. Although polymorphism studies have lost their significance compared to previous years when genetic studies gained popularity, some polymorphisms in the genome have been reported to play considerable role in development of pathological conditions. Their importance in nasopharyngeal carcinoma, osteosarcoma, and pediatric tuberculosis is reported in 3 research articles. Integrated diagnostic approach including clinical and biochemical analysis followed by genetic tests was discussed in children with neurodevelopmental and neurometabolic diseases. Notwithstanding a great deal of advanced genetic testing that has become available, cytogenetics never lost its importance. One of the studies points out that patients benefit from the information that could be provided by cytogenetics in clinical decision-making, followup, and prognosis in myelodysplastic syndrome. On the other hand, fragile X, which is the most common genetic cause in hereditary intellectual disability syndromes, was discussed on a basis of a large number of enrolled subjects. Genetic variant in hepatitis C virus and its effect in the course of antiviral treatment were also evaluated in chronic hepatitis C patients and has made an important contribution to the knowledge of patients' management. Finally and undoubtedly, next generation studies as a robust and cost-effective genetic technology have found place in this special issue in 3 clinical studies relating to the breast and ovarian cancer, Charcot-Marie-Tooth disease, and retinitis pigmentosa.

In summary we aimed to combine reports relating to various genetic technologies in a number of disease conditions. However, as mentioned in the review article by A. A. Durmaz et al., novel technologies in the field of genetics have noticeably accelerated in the last few decades. Even though Moore's law will continuously apply to the developmental speed in computer technologies, the cost of sequencing has declined and the data obtained from NGS platforms has increased at a rate that outpaced Moore's law. Recently a new era has clearly begun with genetics to be one of the major players in human life. In the near future, it will be possible to estimate the risks and prepare prevention strategies for a number of genetically based conditions just by analyzing a few drops of blood. Genetic cloning, genetic engineered organisms, extreme longevity, artificial intelligence, and personalized treatment will become a reality.

*Ozgur Cogulu* *Jacqueline Schoumans* *Gokce Toruner* *Urszula Demkow* *Emin Karaca* *Asude Alpman Durmaz*

